# Seizure triggered by flicker electroretinogram in a patient with no history of epilepsy

**DOI:** 10.1007/s10633-020-09813-9

**Published:** 2020-12-23

**Authors:** Sven P. Heinrich, Hansjürgen Agostini

**Affiliations:** 1 Eye Center, Medical Center, University of Freiburg, Killianstr. 5, 79106 Freiburg, Germany; 2 Faculty of Medicine, University of Freiburg, Freiburg, Germany

**Keywords:** Epilepsy, Paroxysmal event, Photic-induced seizure, Electroretinography, Safety

## Abstract

**Purpose:**

It is well known that repetitive flash stimulation may trigger seizures in susceptible individuals. Nevertheless, reports of such incidents occurring during recording of a flash electroretinogram (ERG) are extremely rare. Here, we describe the case of a photic-induced seizure triggered during an ERG recording in the absence of a history of epilepsy or other paroxysmal events.

**Methods:**

A 14-year-old male patient presented with reduced visual acuity and impaired mesopic vision. Ophthalmological exams confirmed the patient’s complaints but were inconclusive as to the underlying pathophysiology. An ERG recording was performed, during which the 30-Hz flicker stimulus triggered a seizure.

**Results:**

The ERG was essentially normal, with the exception of a 7-Hz rhythm superimposed onto the flicker ERG response that was recorded when the seizure developed.

**Conclusions:**

The present case highlights the possibility that the 30-Hz ERG flash stimulus triggers a seizure in patients with no previous paroxysmal events. Literature evidence suggests that the likelihood of such an incident could be reduced by stimulating monocularly.

## Introduction

Photic-induced seizures are a well-known phenomenon in epileptology. Fisher et al. [1] estimate the prevalence of seizures from light stimuli at approximately 1 per 10,000, with a higher prevalence of 1 per 4000 in the age range of 5–24 years. In particular, rapid sequences of flashes that extend over a large part of the visual field have been reported as an effective trigger stimulus [1]. The relevant frequency range includes 30 Hz, which implies that recording a standard flicker electroretinogram (ERG), as stipulated by the ISCEV ERG standard [2], may pose a potential risk. However, reports on seizures induced by ERG stimuli are scarce, with only one case revealed by a PubMed search.^1^

Hayashi et al. [3] describe the case of a 34-year-old male who presented with reduced vision in one eye for 15 years and more recent night blindness. The patient developed a convulsive seizure during an ERG recording. It was most likely induced by the 30-Hz flicker stimulus, although the on–off stimulus could not be excluded as a trigger.

Here, we report on a case with no known history of epilepsy or other paroxysmal events who experienced a seizure during the recording of the 30-Hz flicker ERG.

## Case description

The 14-year-old male patient presented with complaints of reduced acuity and impaired vision at low light levels. He had moderate myopia (first glasses fitted at 10 years of age) and was referred to our outpatient clinic to exclude a hereditary retinal disease.

Corrected decimal visual acuity was 0.5 in both eyes, increasing to 0.7 in the left eye with a pin hole. Pupils were isocor, and there was no relative afferent pupillary defect. A mild exophoria was present. In the Lang stereo test, the car and star symbols were recognized by the patient. Color vision (Ishihara test) was unimpaired. Kinetic perimetry showed a mild loss of sensitivity. Eye motility was normal with the exception of some end-position nystagmus. The anterior segment was normal. Both papilla were vital, with the right one slightly flatter and the left one tilted. Optical coherence tomography showed normal ganglion cell volume, and fundus autofluorescence imaging was unremarkable. No family history of visual disorders was reported except for myopia.

As no organic correlate of the visual complaints could be identified, another visit was scheduled about 10 weeks later. The mostly unremarkable findings of the initial visit were replicated. Additionally, the subjective complaints of impaired vision at low light levels were confirmed with the Mesotest II (Oculus Optikgeräte GmbH, Wetzlar, Germany), where the patient could not identify any optotypes. Goldmann visual fields showed a mild concentric relative constriction, with the outer limits with target III/4 intact.

Subsequently, at the same visit, a full-field ERG measurement was performed, including all flash strengths recommended by the ISCEV standard [2], using a Q450 Ganzfeld stimulator (Roland Consult GmbH, Brandenburg, Germany) as part of a custom ERG set-up. Pupils were dilated with tropicamide. Because the fiber electrodes that are normally used in our electrophysiology unit were not tolerated by the patient, gold cup skin electrodes were applied. ERG testing was performed binocularly and proceeded normally up to the point where the light-adapted 30-Hz flicker ERG was recorded. During the second pass of the flicker stimulus (each pass lasting for 3 s with several seconds in between), the patient experienced a seizure. The recording of the ERG was discontinued, and the technician together with the accompanying parent attended to the patient and arranged for further assistance. The seizure ceased without specific intervention. There was no loss of consciousness, possibly with the exception of a short moment before the technician opened the light-proof curtain separating her from the patient.

The parent confirmed that this had been the first incident of this type and that she was unaware of any earlier event that would hint towards epilepsy. She furthermore reported no known family history. The patient was subsequently admitted to a pediatric hospital with a specialized neuropediatric service. A complete workup was performed, including magnetic resonance imaging of the head with contrast agent and diffusion imaging, albeit without relevant findings.

**Fig. 1:**
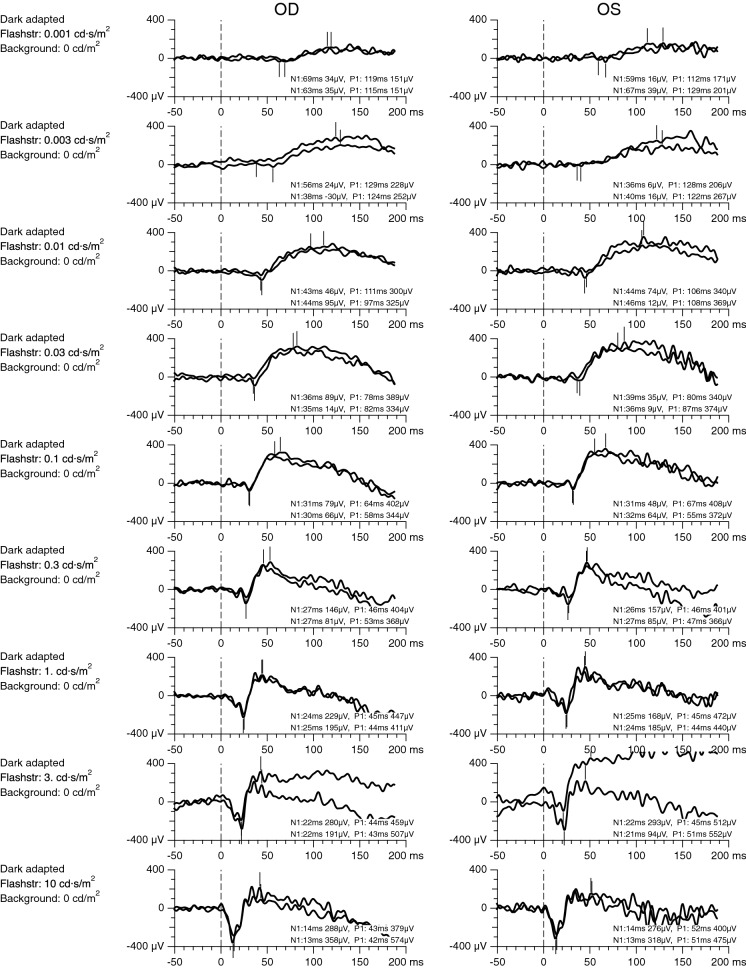
Scotopic ERGs for flash strengths ranging from 0.001 cd/m^2^ (top) to 10 cd/m^2^ (bottom). Recordings were performed with skin electrodes, and amplitudes were scaled to approximately match recordings with fiber electrodes

**Fig. 2:**
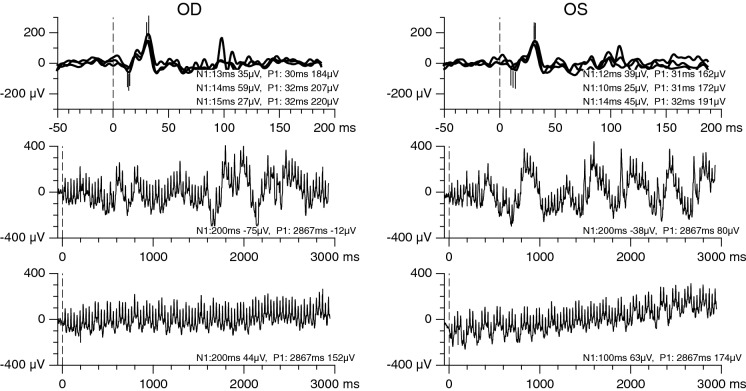
Photopic single-flash ERGs (top) and two recordings of 30-Hz flicker ERGs (middle and bottom). Recordings were performed with skin electrodes, and amplitudes were scaled to approximately match recordings with fiber electrodes. All responses were in the normal range or, in the case of the flicker response in right eye, slightly supranormal. A 7-Hz rhythm is present in the second recording of the flicker ERG. In contrast, normal movement artifacts are only present in the first recording. Note the different abscissal scaling of the single-flash and flicker ERGs

The ERG recordings were mostly within the normal range, when accounting for the use of skin electrodes (scotopic ERGs, Fig. 1; photopic ERGs, Fig. 2). The only exception is a slightly supranormal flicker response in the right eye and a superimposed slow oscillation (approximately 7 Hz) in the second pass of the flicker ERG.

As no cause of the visual impairments could be identified, the patient was preliminarily advised to continue observing his visual status and to have regular ophthalmological checks. He did not experience any further seizures, as confirmed by a follow-up phone call five months after the incident.

## Discussion

In the presented case, the patient experienced a seizure while being exposed to the 30-Hz ERG flicker stimuli. It seems very unlikely that this occurred coincidentally; rather, it appears safe to assume that the visual stimulus triggered the paroxysmal event. This is reminiscent of the so-called Pokemon incident, where a sizeable number of children watching a cartoon movie on television developed a seizure during a scene with intense repetitive flashes [4]. The present patient is in the typical age range for a first-time occurrence of a photic-induced seizure [5].

We tentatively interpret the low-frequency oscillation visible in the second flicker ERG recording as an early sign of the seizure. However, it is not necessarily a direct electrical effect of seizure-related cortical discharges. Rather, it may be an indirect result of eyeball movement or eyelid and forehead jerks.

At present, we hesitate to interpret the supranormal flicker ERG responses in the right eye as a sign of abnormal retinal function, given that the ERG was recorded with skin electrodes and the conversion of ERG amplitudes obtained with skin electrodes into values that are numerically equivalent to those obtained with fiber electrodes is only approximate.

There is evidence that the likelihood of seizures can be substantially reduced by exposing only one eye at a time to photic stimulation [1]. This is in principle a feasible solution for ERG recordings, albeit at the expense of longer testing durations. In the past, we have already used monocular stimulation in some patients with a history of seizures in the absence of known photosensitivity after discussing the issue with the patient. However, in most cases with known epilepsy, we have refrained completely from recording the flicker ERG.

The present case was the only seizure that has occurred in our electrophysiology unit within a time span of at least 40 years, probably the only one ever (M. Bach, personal information, March 2020), i.e., the only such incident among many thousand ERG recordings. However, given the well-known phenomenon of photic-induced seizures, the nearly complete lack of reports on such incidents is nevertheless surprising. We suspect that there are more such cases than those described in the extant literature.

Interestingly, both the present patient and the patient in the other reported case of a seizure induced during an ERG recording [3] complained about visual impairments under low light conditions. Certain types of seizure-associated disorders such as sialidosis type 1 and benign adult familial myoclonic epilepsy may coincide with night blindness [6, 7], which increases the likelihood of such patients presenting to an ophthalmic clinic and an ERG being ordered. However, we did not find any reports of these specific disorders being linked to a higher incidence of photosensitivity.

The processes underlying a photic-induced paroxysmal event are not fully understood. At least for pattern-induced seizures, a cortical origin is implied by the differential effectiveness of various combinations of binocular stimulation as a trigger [8]. More specifically, changes in the balance of excitatory and inhibitory processes have been suggested as an important factor [9].

In summary, the present case highlights the possibility of a seizure being triggered by the 30-Hz ERG flicker stimulus. The question arises whether patient groups with an increased likelihood of developing a seizure could be identified in clinical practice. In these patients, the flicker ERG could then be omitted or, if its recording is considered essential, the likelihood of a seizure could be reduced by using monocular stimulation.

## Footnotes

1 A PubMed search was performed on November 27th, 2020, using the query “electroretinogram AND (epilepsy OR seizure).” The titles of the search results were screened, and, when the respective title was inconclusive, the abstract and/or the full texts were checked. Only one pertinent article was found.
